# Risk assessment of hollow-bearing trees in urban forests

**DOI:** 10.1038/s41598-023-49419-0

**Published:** 2023-12-14

**Authors:** Marzena Suchocka, Tomasz Jelonek, Magdalena Błaszczyk, Marzena Wińska-Krysiak, Marcin Kubus, Maciej Ziemiański, Hazem M. Kalaji

**Affiliations:** 1 Department of Landscape Architecture, Institute of Environmental Engineering, Warsaw University of Life Sciences-SGGW, Nowoursynowska St. 159, 02-776 Warsaw, Poland; 2 Faculty of Forestry and Wood Technology, Poznan University of Life Sciences, St. Wojska Polskiego 28, 60-637 Poznan, Poland; 3 Department of Plant Protection, Institute of Horticultural Sciences, Warsaw University of Life Sciences-SGGW, Nowoursynowska St. 159, 02-776 Warsaw, Poland; 4 Department of Landscape Architecture, West Pomeranian University of Technology, Papieża Pawła VI St. 3a, 71-459 Szczecin, Poland; 5 Faculty of Biology, University of Warsaw, Warsaw, Poland; 6 Institute of Technology and Life Sciences-National Research Institute, Al. Hrabska 3, Falenty, 05-090 Raszyn, Poland; 7 Department of Plant Physiology, Institute of Biology, Warsaw University of Life Sciences-SGGW, Nowoursynowska St. 159, 02-776 Warsaw, Poland

**Keywords:** Environmental sciences, Forestry, Urban ecology

## Abstract

The paper is a study of risk assessment posed by trees in selected urban woodlands (urban forests) of Warsaw. Two groups of trees were analysed and compared: exhibiting signs of maturity and ageing (hollow-bearing trees with open or hidden cavities and/or caries) and with no signs of decay. 373 individual trees growing near routes frequently or continuously used for recreational purposes were examined using Roloff's vitality classification, and tree risk assessment method, complemented by instrumental studies: a resistance resistograph, pulling tests, and sonic tomography (SoT). The collected data was analysed using the Chi-square test. The results indicate that it is not possible to conclude unequivocally that the presence of hollows in aged trees significantly increases the risk of falling. According to the safety factor results from the SoT and pulling tests, no correlation was demonstrated between the presence of hollow trees and an increase in risk class. The highest proportion of hollow trees (89.42%) was in the low risk group for trunk fracture and uprooting. The results also indicate the coherence of the diagnostic methods to be necessary for providing sufficient information to assess the statics and, ultimately, as our study showed, the protection of hollow trees.

## Introduction

Cities are complex socio-ecological systems that provide a habitat for an increasing population, projected to rise by an additional 2.5 billion inhabitants by 2050^1^. Urban forests play a crucial role in ensuring suitable living conditions for city residents by delivering a variety of tree-based benefits in the form of ecosystem services, including air purification, carbon sequestration, noise reduction, temperature control, and flood control, all essential in the face of climate change^2,3^. These forests also offer a range of cultural, social, psychological, and aesthetic values, such as stress relief and social cohesion, thus enhancing overall wellbeing^4^. Most of these ecosystem services are public, available to everyone at no charge^5^.

The growing recognition of the significance of urban forests is mirrored in the increasing number of policies, initiatives, programmes, and documents dedicated to global sustainability. For instance, the United Nations Economic Commission for Europe emphasises that Sustainable Urban and Peri-urban Forestry (SUPF) is a strategic nature-based solution to create green, sustainable, and resilient cities. SUPF requires long-term urban forest ecosystem management to ensure urban trees and forests are well-cared for and mature, optimising their benefits over time^6^.

The quantity and quality of ecosystem services directly correlate with the quantity, quality, and distribution of urban forests^4,7^. The smallest constituent of an urban forest is an 'isolated tree', and the largest is an 'urban woodland'^8^. This paper specifically focuses on woodlands, given their broad impact on cities and their inhabitants and their potentially rich and diverse structure. In Polish, and many other European languages, the term 'urban forest' pertains more to forests or forest ecosystems than to street or park trees^9^. Therefore, in this context, 'urban forest' refers to 'woodlands' within a city.

Urban environments are generally unfavourable for tree growth and development due to multiple stress factors like drought, air pollution, concrete and impermeable surfaces, soil compaction, restricted root growth space, and mechanical damages. As a result, trees in cities, particularly street trees, typically 'live fast and die young'^10^. This leads to city centres being dominated by young, undersized trees that provide limited benefits to city dwellers^11^. In contrast, urban forests offer a unique, nurturing habitat for trees, providing a reservoir of large and often mature trees that are particularly valuable and desired in cities amid climate change^12^. The habitat in which a tree grows is vital to its longevity and its ability to achieve maximum lifespan and growth^13^. In Europe, large and old trees, also known as veteran trees, are generally found in three ecosystems: remnants of orchards or traditionally managed forest zones, old-growth forests, or parks^14^.

Large and ageing trees are essential structures for biodiversity conservation. Owing to their size, they are crucial providers of resources to a diverse array of species^15,16^, including mammals^17^, birds^18^, reptiles^19^, invertebrates^20^, mosses, lichens, fungi ^21^, and others. They can shelter many endangered specialised species of flora and fauna^14^. As a tree ages, it begins to develop unique physical attributes, including ecological niches and microhabitats, such as hollows. Such a tree can be referred to as a habitat tree (serving as a host of a microhabitat) or a hollow-bearing tree^14,15^. Its ecological value typically increases with its diameter and bark thickness, and thus with tree age^14^. This cannot be provided by younger trees^22^. Therefore, removing a habitat tree results in an inability or highly limited potential to replace it in the short term. This raises the risk of extinction of hollow-dependent species^23^.

Unfortunately, hollow-bearing trees are at a high risk of being cut down due to potential safety hazards they pose to the public and infrastructure^24^. Concerns mostly relate to structural failure leading to falling branches or tree toppling, which can pose a significant threat to human safety^25,26^. In fact, the presence of a hollow does not signify a threat, and a safe tree can be almost entirely hollow^22^. During their growth, trees optimize the woody tissue for both mechanical and physiological functions (so-called adaptive growth)^27,28^. Only three factors are responsible for the mechanical weakening of the wood structure: sudden trauma, the development of decay, and/or physical or chemical changes^29^. The presence of a hollow does not indicate the need for tree removal^22^.

However, field observations have shown that the presence of a hollow, especially when externally detectable based on cavities, often leads to the tree being considered unsafe, more so than trees with partially decayed wood and reduced mechanical strength^25^. Practitioners involved in tree assessment face challenges in balancing the decision to leave the tree and potentially observe it further with public pressure to remove it^15,16^. Therefore, they may make emotional decisions for a variety of reasons^30^, which can lead to discrepancies in the recommendations made, keeping potential responsibilities in mind^31^. However, the decision on risk classification depends on expert knowledge and experience^32,33^. Experts have several technical methods at their disposal for assessing tree vitality, including a resistance recording drill, a pulling test to test the root system, SoT, and visual risk assessment methods^34^. Visual methods, which are to some extent qualitative and subjective, and at the same time probably the most commonly used by arborists, often fail to convince decision-makers to leave a tree^22^. Therefore, there is a need for studies conducted with a similar methodology but with the support of dedicated devices that allow for a less subjective risk assessment of these high-value hollow bearing trees.

The aim of this study is to compare the level of risk posed by trees exhibiting signs of maturity and ageing, particularly the presence of visible and hidden cavities and decay, with trees lacking these signs that are growing in the natural environment of urban forests under reserve protection. The results of this study are expected to address the research gap concerning hollow-bearing trees in urban woodlands and enhance the management of these trees in the risk assessment process.

### Research questions

The main research questions were:

- Does the risk class (A, B, C, and D) depend on the presence of hollows or decay?
- Is there a correlation between the presence of hollow cavities and decay in aged trees and the Roloff’s scale adopted in the assessment?
- Is the height safety factor in the SoT study related to the risk scale of hollow trees and trees without cavities or decay?
- Do hollow trees and trees with decay have a higher safety factor value on average, including for load tests? What are the average values for trees with and without hollows?
- Do the biometric characteristics of trees with and without hollows and/or decay differ?

## Methods

### Study area

**Figure 1:**
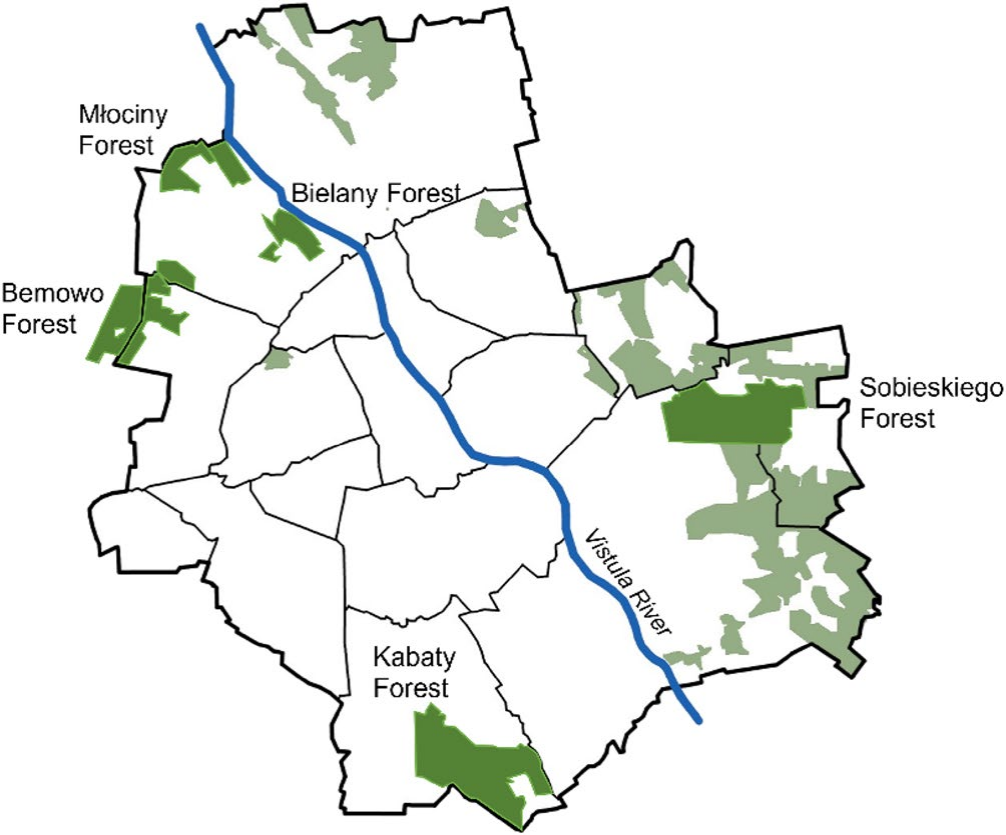
The location of the site areas (five municipal forests) on the territory of Warsaw^35^; Source: own elaboration (created with Vectorworks 2023 software).

According to 2020 data, Warsaw's urban forests cover about 7968 hectares, which is about 15% of the city's area. They consist of 27 complexes, 15 of which are under the management of the City of Warsaw. Some of the forests have reserve status. Five urban forests were the site of the research presented in this article: Młociny Forest, Bielany Forest, Bemowo Forest, Kabaty Forest, and Sobieskiego Forest. The location of the studied trees can be seen in Fig. 1.

### Tree sample

All the trees included in the study were large and ageing, with trunk circumferences ranging from 107 to 527 cm, measured at 130 cm above the ground. They were located within an area defined by the potential range of damage that could be caused by a tree falling, which is 1.5 times the height of each tree. The study focused on trees situated near routes that are frequently or continuously used for recreational purposes. These locations were selected due to the heightened risk they pose to visitors. According to standard procedures, trees in these areas should be inspected more thoroughly and more frequently than those in less frequented areas. This protocol is applied to every tree^36^. Tree data was collected in the summer of 2021. The database included 384 trees, with 112 trees located in Młociny Forest, 115 in Bielany Forest, 35 in Bemowo Forest, 37 in Kabaty Forest, and 85 in Sobieskiego Forest. Out of these, 373 individuals were selected for a comprehensive analysis. The sample included two main tree species: *Tilia cordata* Mill. (n = 91), and *Quercus robur* L. (n = 257), along with nine other species (ranging from one to eight specimens), including 104 (27.9%) with visible hollows. A detailed list of the tree species included in the survey is available from the authors of the article. Mean and median circumferences of the trees are presented in Table 1.

**Table 1:** Descriptive statistics of the perimeter of the studied trees.

Hollow	Trunk circumference (cm)				
	Mean	Stand dev	Q25	Median	Q75
No	262.58	65.50	221.00	261.00	295.00
Yes	280.12	66.00	234.50	274.50	323.00
Total	267.57	66.03	227.00	263.00	305.00

### Research involving plants

As our study covered field study observations and non-invasive measurements of trees without collecting plant material, no permissions were needed in compliance with the IUCN Policy Statement on Research Involving Species at Risk of Extinction and the Convention on the Trade in Endangered Species of Wild Fauna and Flora.

### Tree vitality assessment

Each tree was evaluated according to Roloff's^37^ visual classification and based on the vitality of the distal parts of the crown. Trees were categorised into four groups: R0 refers to trees in a phase of intense shoot growth; R1 refers to trees with slightly delayed shoot growth; R2 refers to trees with visibly delayed shoot growth; and R3 refers to trees with no possibility of regeneration and no likelihood of reverting to the R2 class.

### Risk assessment

**Figure 2:**
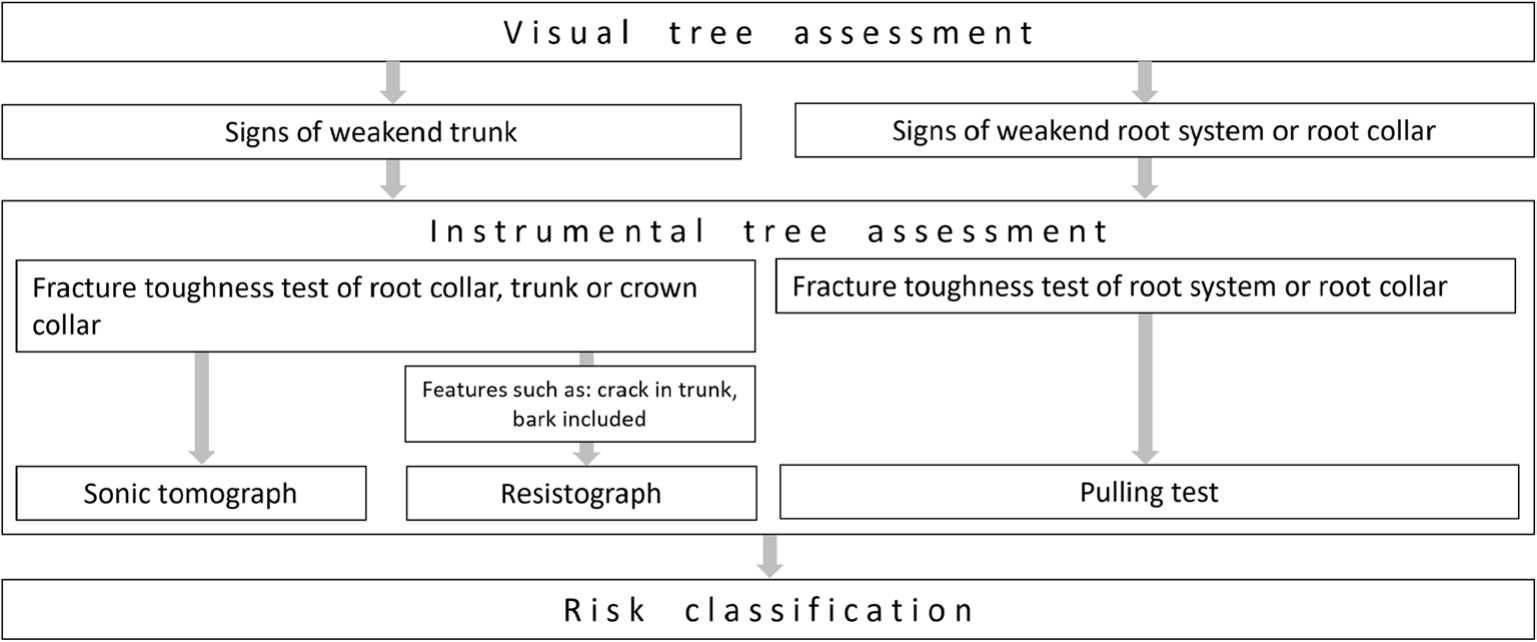
Framework for selection of risk assessment methods.

A standard procedure was used to determine the level of risk associated with the trees under study—the selected tree risk assessment method included a combination of ISA/BMP and TRAQ^38^ methods.. Mature and aged trees growing in the vicinity of footpaths and roads have been selected for the study. The criterion for the selection of trees for risk assessment was their location in the vicinity of the routes at a distance of less than 1.5 tree heights. Initially, noticeable defects were looked for while examining the overall vitality of the tree. Then, a more thorough examination of these defects was conducted^39^. The zone of decay inside the tree trunk was identified on the basis of visible hollows (open cavities) or after examination with a diagnostic hammer (hidden cavities and decay).To obtain any missing information needed for the classification of risk classes in relation to tree statics, instrumental tests were performed using specialised equipment when necessary. These tests involved using a resistance drilling (PowerDrill F 400, IML), sonic tomograph (ArborSonic 3D, Fakopp Enterprise Bt.), or a pulling test (Pulling Test, Fakopp Enterprise Bt.)^40–43^. The selection of the test method for each tree and the interpretation of the results were carried out by an experienced expert, chosen preferably as the weakest zone of the investigated tree. Tree assessor was deciding on instrument selection based on limitations of each instrumental investigation methods^34,44,45^. If there were doubts about the mechanical strength of the root collar, trunk or crown collar, a SoT test was performed. In the case of features that prevented SoT testing, such as bark included or cracks in the trunk, a resistance drilling was performed. In case of doubts about the mechanical strength of the root system or root collar, a static pulling test was performed (Fig. 2).

### Instrumental investigation

**Figure 3:**
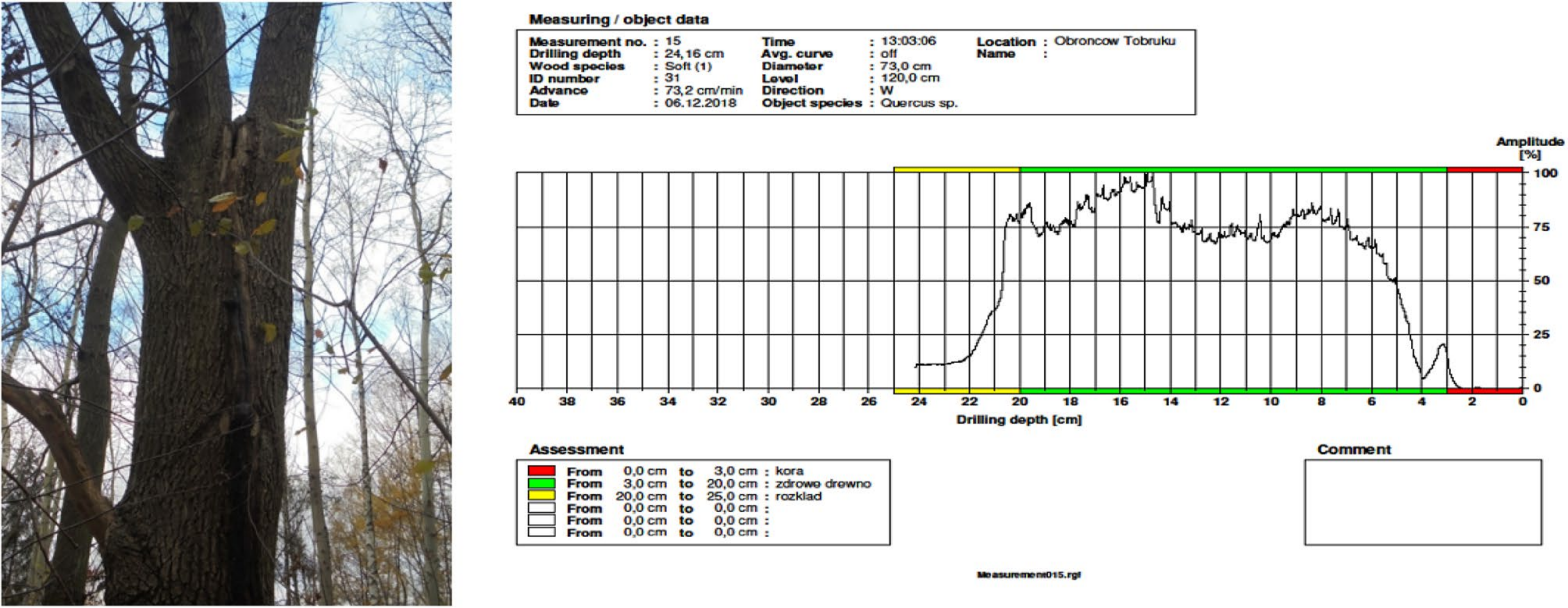
Hollow tree, *Quercus robur* L., with bark included on trunk, tested with resistograph, Bemowo Forest, tree no 31.

To examine the internal structure of the trunks of selected trees, we used a resistance drilling measurement. This method measures the resistance encountered by a 3 mm diameter drill bit as it penetrates the wood. By simultaneously recording the drilling resistance and the speed of the needle, early and otherwise hard-to-detect irregularities in the wood structure could be identified. The feed and drill speed can be adjusted from 15 to 200 cm/min and from 1500 to 5000 rpm, respectively. Each drilling location was chosen to be free of visible wood defects, such as knots or cancers. The main variable studied using the resistance drill was the Resi Amplitude (RA%), which quantifies the resistance that the wood presents to a drill bit moving at a specified constant speed, examined at 0.1 mm increments from 0 to 100%. The maximum and minimum RA data (RAmax and RAmin) were then extracted using dedicated software (Fig. 3).

**Figure 4:**
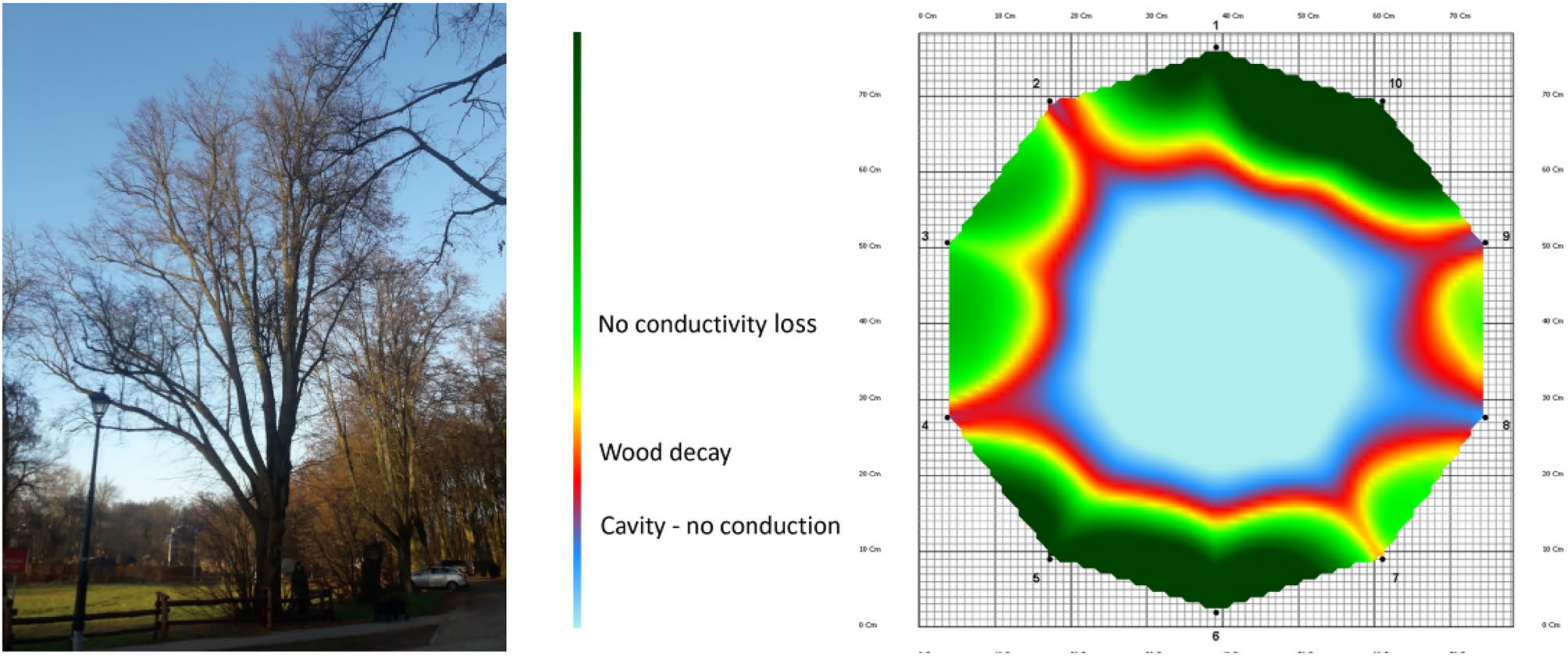
Hollow tree (*Tilia cordata* Mill.), Młociny Forest, tree no 66—SoT test result.

Selected trees were examined using SoT at predetermined heights—the weakest zone of the trunk was determined by visual examination. This produced a digital map of wood density (tomogram) in the stems of the living trees. After entering additional data, the mechanical break strength of the tree was determined at the SoT level. The following thresholds for fracture strength were established: high fracture strength (151% and above), moderate fracture strength (101–150%), and no fracture strength (0–100%) (Fig. 4). CT scans were repeated (multiple scans) when additional information was needed—to determine the extent of the hollow trunk section or decay distribution in case of ambiguity. Each time, the height of the test was documented. Tests were performed in accordance with the manufacturer's instructions and took into account the height of the tree, crown shape, and species, among others. The wood strength of the test species was entered into the software during the measurement based on the Stuttgart table of wood strength^46^.

To determine the safety factor and assess the overall strength of the tree, dynamic static root strength tests and a pulling test were used (Supplementary Information).

For many years, winching tests or pulling tests have been used during research in tree biomechanics as a means to determine the resistance of trees against rupture and uprooting.

Usually, the tests are designed to cause ultimate failure and therefore lead to the destruction of the subject trees. In practical arboriculture, a non-destructive assessment of tree risk is required in order to identify hazard trees and to be able to retain mature valuable trees. Often, veteran trees will be sufficiently stable despite obvious defects in their wooden body. Therefore, static load tests were developed in the mid 80’s at the University of Stuttgart in Germany and were applied on more than 10.000 trees so far in Europe and North America.

The actual measurements draw a very accurate picture of the trees' reaction to loads. But the aim of every investigation is to determine the level of risk involved in retaining the subject tree or to reliable prove the need for its removal. Using this methodology to assess the likelihood of failure (stem breakage or uprooting), the subject tree is pulled to simulate moderate wind loading and the resultant changes in fibre length and root plate inclination are measured. The experiment is designed to determine how the tree responds to defined loads.

While the tree is exposed to increasing load, its stem bends and its root plate tilts to a miniscule degree that is invisible to the human eye. High sensitivity instrumentation continuously monitors the tree reaction and links it to the applied load. Data is logged via radio into a computer and is being stored electronically for later evaluation.

The tree is tugged using a stable anchor point with a force of 10–40 kN, simulating a wind pressure force of 33 m/s^47^.

The pulling force is monitored using an electronic forcemeter (dynamometer) with a resolution of 0.01 kN (roughly 2.2 lbf). The bending of the tree stem is detected via high-resolution displacement transducers (inclinometers) that monitor fibre strain at an accuracy of 0.001 mm. At the same time, the inclination root crown just above ground is detected by highly sensitive inclinometers (resolution 0.001°). Every test will be terminated at very low reaction levels in order to ensure that deformations are fully reversible and the subject tree remains structurally undamaged.

The following thresholds for root and root collar fracture strength were established: fracture strength (151% and above), moderate fracture strength (101–150%), and no fracture strength (0–100%).

The results from tomography may be unreliable due to structural damage and defects in the tree, such as fused logs, frost cracks, and embedded bark^48^. Each test result was cross-checked by visual inspection, and any with unreliable results were excluded from further investigation.

The objective results of the instrumental tests (safety factor), including the probability of stem fracture assessed with the SoT and the risk of tree uprooting assessed with the pulling test, were analysed for trees with and without visible cavities. We made the following assumptions regarding safety factors: over 150% strength signifies low risk, 100 to 150% signifies moderate risk, and up to 100% signifies high risk. We conducted a statistical analysis of the mechanical strength scores (%) obtained from the tomograph and pulling test, for all the trees studied, comparing the scores obtained for trees with hollow trunks and trees with solid trunks.

According to expert knowledge and experience^33^, the total number of trees in the study sample was divided into risk classes adapted from the TRAQ system^49^. As a result of deep visual and instrumental investigation the studied trees were classified into the following risk classes: low (A), moderate (B), high (C), and extreme (D)^22,38^.

Analyses were conducted for a group of trees with signs of wood decomposition (hollow bearing trees with open or hidden cavities and/or caries) and tree group with no signs of decay on the trunk.

### Presence of hollow trees and trees with caries in combined instrumental safety factors categories

The analyses of the relationship between the safety factor categories, as determined instrumental measurements has been conducted for hollow bearing trees and trees with cavities. Occurrence of hollow trees in different safety factors categories derived from instrumental measurements (without resistance drilling) has been categorised as: 0 high risk of stem fracture, 1 low risk of stem fracture and uprooting, 2 moderate risk of stem fracture and uprooting, 3 high risk of uprooting.

### Biometric parameters analysis

Biometric traits of trees with and without hollows were analysed: Diameter at Breast Height (DBH), tree height (m), crown width (m).

### Statistical data analysis

The data analysed in the paper were primarily quantitative. These were trees with and without hollows, which, based on different assessments, were classified into different grades related to the risk of their failure. Qualitative analyses were also carried out, relating to the relationship between biometric traits of trees such as DBH, tree height, and crown width in trees with and without hollows. Conducting these types of analyses simultaneously allows for a more comprehensive examination of the relationships and interactions that occur, broader conclusions, and thus a holistic description of the study area.

Since the data analysed are primarily quantitative in nature, being the numbers of trees with and without hollows in different risk classes determined by different methods, statistics based on multivariate tables, which describe the distribution of observations due to several characteristics simultaneously, were used to describe them. We were primarily interested in analysing the effect of the presence of hollows in trees on the assessment of their risk of failure determined by several methods. A Chi-square independence test was used to test the relationship between the two nominal (categorical) variables. The Chi-square independence test assesses whether the observed distribution (here, risk category) depends on tree hollows. In the chi-square analysis, Cochran's condition was met, and the hypotheses stated are that the variables are independent. The paper does not include tables with expected and observed data not to increase its volume. Only the results of the Chi-square test are presented.

Analysis of variance (ANOVA) was used to compare biometric traits of trees with and without hollows, and Spearman rank correlation coefficients were used to analyse relationships:

$$rs=1-\frac{6{\sum }_{{\text{i}}=1}^{{\text{n}}}{d}_{i}^{2}}{\mathrm{n }({n}^{2}-1)}$$

${d}_{i}^{2}$  is the squares of the differences between the ranks of the corresponding feature values ${x}_{i}$ and ${y}_{i}$, n is the number of data pairs.

All statistical analyses were conducted at a significance level of p ≤ 0.05. Statistical analyses were conducted in software STATISTICA 13.5.

## Results

### Risk category vs. hollow trees

**Figure 5:**
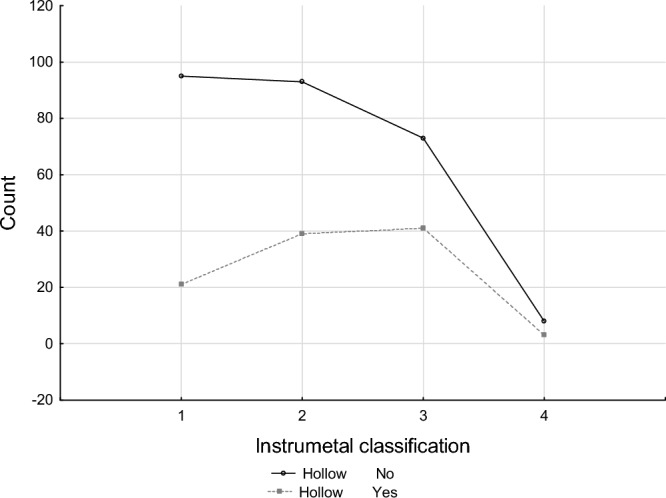
Number of hollow trees in risk categories.

The distribution of hollow trees across each risk category (A, B, C, and D) was analysed. Based on visual assessment, the risk categories comprised of A: 116 trees, B: 132 trees, C: 114 trees, and D: 11 trees (Table 2, Fig. 5). Only three trees with hollows fell into category D, representing less than 2.88% of the total population of hollow-bearing trees. Category A included 20.19%. The small percentage of hollow trees in the highly dangerous category (D) represents trees to remove due to the high risk level.

**Table 2:** Occurrence of hollow trees in different risk categories derived from visual risk assessment method.

Hollow	Risk classes				
	A	B	C	D	Row
No	95	93	73	8	269
% column (%)	81.90	70.45	64.04	72.73	
% row (%)	35.32	34.57	27.14	2.97	
% overall (%)	25.47	24.93	19.57	2.14	72.12
Yes	21	39	41	3	104
% column	18.10	29.55	35.96	27.27	
% row	20.19	37.50	39.42	2.88	
% overall	5.63	10.46	10.99	0.80	27.88
Total	116	132	114	11	373
% overall (%)	31.10	35.39	30.56	2.95	100.00

The independence analysis did not reveal a relationship between the risk categories determined by visual assessment (supplemented by instrumental measurements) and the number of hollow trees in these categories (Table 3). The majority of mature trees with hollows fell into categories C and B, with 41 (39.4%) and 39 (37.5%) trees respectively, accounting for over 21% of the total number of trees surveyed. This finding indicates that the presence of hollows in mature trees does not directly influence its stability as determined by the risk level. Given the noticeable trend of increasing numbers of hollow trees from categories A to C, the presence of hollows should be considered during safety assessments around trees as a factor that could influence tree stability but does not definitively determine it.

**Table 3:** Test of Chi-square independence of the risk determined from visual assessment method with the number of hollow trees in these categories.

	Chi-square	df	p
Chi^2^ Pearson’s	9.4039	3	0.0244
Chi^2^ NW	9.7205	3	0.0211

### Roloff’s scale vs. hollows

**Figure 6:**
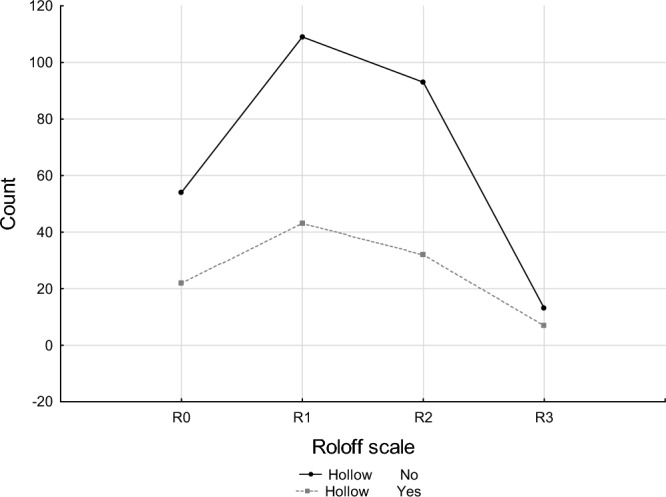
Number of hollow trees in Roloff's classes.

The relationship between the occurrence of hollows in aged trees and their categorisation on the Roloff’s scale was examined. Of the total number of 104 hollow trees across the study population, only 22 trees (21.2%) fell into the R0 category, 43 (41.4%) into the R1 category, 32 trees (30.8%) into the R2 category, and seven trees (6.7%) into the R3 category (Table 4, Fig. 6). Simultaneously, in both the R0 and R1 classes, the percentage of hollow trees in relation to the total number of trees in the group was very similar, slightly over 28%. The greatest quantity of hollow trees, as many as 65 (72.1%), fell into the R1 and R2 categories, representing 20.1% of the total number of surveyed trees (Table 4, Fig. 6).

**Table 4:** Occurrence of hollow trees in different Roloff’s classes.

Hollow	Roloff scale				
	R0	R1	R2	R3	Row
No	54	109	93	13	269
% column	71.05	71.71	74.40	65.00	
% row	20.07	40.52	34.57	4.83	
% overall	14.48	29.22	24.93	3.49	72.12
Yes	22	43	32	7	104
% column	28.95	28.29	25.60	35.00	
% row	21.15	41.35	30.77	6.73	
% overall	5.90	11.53	8.58	1.88	27.88
Total	76	152	125	20	373
% overall	20.38	40.75	33.51	5.36	100.00

However, there was no significant relationship found in the abundance of trees with hollows within each Roloff’s class (p > 0.05). This finding is significant as it suggests that the occurrence of hollows in aged trees does not directly correlate with their vitality as determined by the Roloff’s scale (Table 5).

**Table 5:** Test of Chi-square independence between the incidence of aged hollows in trees and the Roloff’s scale.

	Chi-square	df	p
Chi^2^ Pearson’s	0.8831	3	0.8295
Chi^2^ NW	0.8658	3	0.8337

### Presence of hollow trees in instrumental safety factors categories

**Figure 7:**
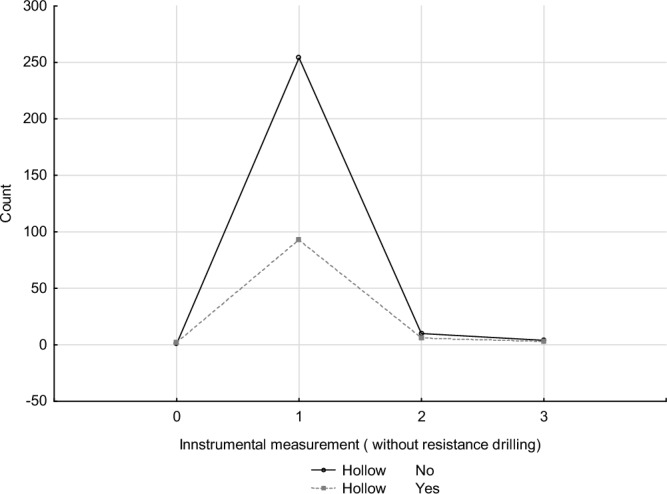
Number of hollow trees in risk categories derived from instrumental measurements (without resistance drilling).

The analysis of the relationship between the safety factor categories, as determined by instrumental measurements, and the number of hollow trees within these categories, showed no statistically significant correlation (Table 6). The largest number of aged trees with hollows were found in category 1 (low risk of trunk fracture and uprooting), accounting for as many as 93 trees. This represented 89% of the hollow trees and 25% of all trees surveyed. This result suggests that the presence of hollows in aged trees does not directly impact their stability as determined by tomograph measurements and load test results. Similarly, trees without hollows were largely represented in group 1, with 94% of all trees with sound trunks falling into this category. It is noteworthy that there were more trees without hollows than with hollows in categories 2 (moderate risk of stem breakage and uprooting) and 3 (high risk of uprooting) (Table 7, Fig. 7).

**Table 6:** The Chi-square test of independence between risk categories determined from instrumental measurements and the number of hollow-bearing trees and trees with cavities in those categories showed no statistical relationship.

	Chi-square	df	p
Chi^2^ Pearson’s	3.962614	3	0.26553
Chi^2^ NW	3.557566	3	0.31337

**Table 7:** Occurrence of hollow trees in different safety factors categories derived from instrumental measurements (without resistance drilling) (0 high risk of stem fracture, one low risk of stem fracture and uprooting, 2 moderate risk of stem fracture and uprooting, 3 high risk of uprooting).

Hollow	0	1	2	3	Row
No	1	254	10	4	269
% column (%)	33.33	73.20	62.50	57.14	
% row (%)	0.37	94.42	3.72	1.49	
% overall (%)	0.27	68.10	2.68	1.07	72.12
Yes	2	93	6	3	104
% column	66.67	26.80	37.50	42.86	
% row	1.92	89.42	5.77	2.88	
% overall	0.54	24.93	1.61	0.80	27.88
Total	3	347	16	7	373
% overall	0.80	93.03	4.29	1.88

### Hollow-bearing trees and instrumental investigation safety factor results—comparison to trees without hollows

**Figure 8:**
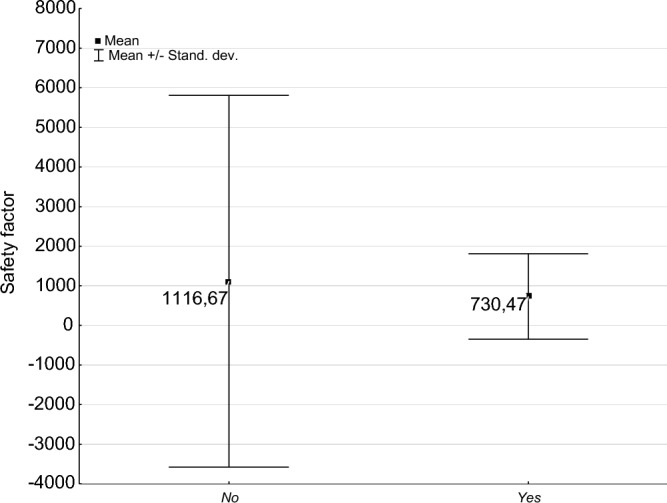
Statistical characteristics (measures of location and dispersion) of safety factor (SoT) of hollow-bearing trees with and without cavities.

The average safety index [%] obtained from the SoT and uprooting test for all evaluated trees was 984%. For trees without hollows, the SoT safety index was 1117%, while for trees with hollows, it was 730% (Table 8, Fig. 7). Even though there is a substantial difference in the safety index between trees with and without hollows, the variance analysis (p ≤ 0.05) revealed no significant difference in the safety index between these two groups of trees (Table 9). There were significant differences in dispersion and location of safety index (SoT) values between trees without hollows (large dispersion) and trees with hollows (small dispersion). The broad dispersion of results in the case of trees without hollows likely led to the lack of statistical differences (Fig. 8).

**Table 8:** Characteristics of the safety factor and load test in aged trees with hollows and trees without hollows.

Hollow	Mean	N	SD	Min	Max
SoT safety factor					
No	1116.67	147	4692.80	16.00	51,200.00
Yes	730.47	77	1080.87	28.00	8685.00
Total	983.91	224	3853.59	16.00	51,200.00
Pulling test safety factor					
No	107.31	110	13.41	101.00	149.00
Yes	114.00	37	14.69	101.00	145.00
Total	109.00	147	14.00	101.00	149.00

**Table 9:** Analysis of variance of the SoT safety factor and pulling test safety factor for trees with and without hollows.

	SS	df	MS	SS	df	MS	F	p
SoT safety factor	7,536,724	1	7,536,724	3.3041E + 09	222	14,883,122	0.5064	0.4775
Pulling test safety factor	1236	1	1236	2.7380E + 04	145	189	6.5464	0.0115

**Figure 9:**
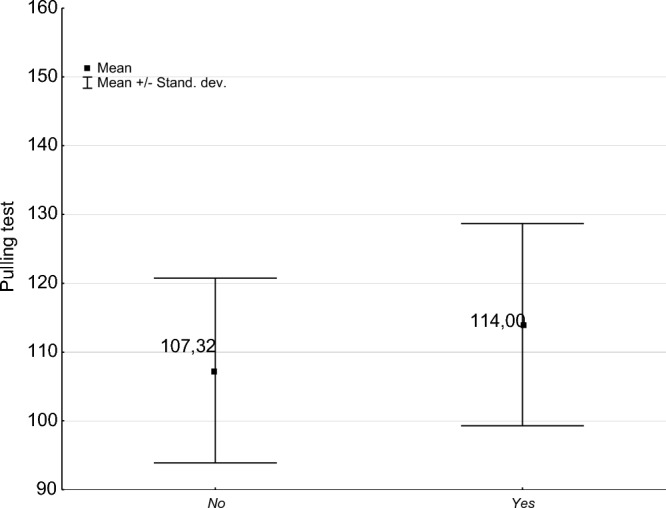
Statistical characteristics (measures of location and dispersion) of the pulling test of hollow-bearing trees with and without cavities.

For the uprooting test, the average values for trees with and without hollows were not similar (114% for trees with hollows and 107% for trees without hollows). However, due to the low dispersion of results, there were significant statistical differences between the groups (Table 9, Fig. 9). Trees with hollows showed a statistically significantly higher uprooting test value (114%) than trees without hollows (107%).

### Biometric traits

An evaluation of the biometric features of trees with and without cavities was conducted. Trees that had hollows exhibited an average diameter at breast height (DBH) of 83.6 cm, a height of 21.98 m, and a crown width of 15.35 m. Conversely, trees without cavities had an average DBH of 89.2 cm, a height of 22.52 m, and a crown width of 13.75 m (Table 10). An analysis of variance indicated that there were significant statistical differences (p ≤ 0.05) in DBH and crown width between trees with and without cavities and hollows, but there was no significant difference in tree height (Table 11). Trees with hollows were significantly thicker and had narrower crowns.

**Table 10:** Characteristics of biometric traits of trees with and without cavities or caries.

Hollow	DBH (cm)		Tree height (m)		Crown width (m)	
	Mean	SD	Mean	SD	Mean	SD
Yes	83.6	20.86	21.98	3.64	15.35	4.22
No	89.2	21.02	22.52	3.48	13.75	3.70
Total	85.2	21.03	22.14	3.60	14.90	4.14

**Table 11:** Analysis of variance of biometric traits of trees with and without cavities or caries.

	SS	df	MS	SS	df	MS	F	p
DBH (cm)	2320.749	1	2320.749	159,084.6	364	437.046	5.310	0.0218
Tree height (m)	21,324	1	21.324	4745.9	367	12.932	1.649	0.1999
Crown width (m)	189.893	1	189.893	6087.4	365	16.678	11.386	0.0008

This study scrutinised the relationships between the biometric characteristics of trees and the safety factors derived from different measurements and tests. Statistically significant associations were found between the safety factors (instrumental) and tree crown width. A significant correlation was also observed between safety factor categories and tree height, DBH, and trunk circumference. Resistance drilling showed a positive correlation with crown width, whereas the pulling test exhibited a negative correlation with crown width and a positive correlation with tree height (Table 12).

**Table 12:** Spearman rank order correlation for selected variables (p ≤ 0.05).

Correlations for variables	Safety factor (instrumental)	Safety factor categories	Resistance drilling (cm)	Required minimum wall thickness of healthy wood (cm)	Pulling test
Crown width (m)	−0.1731	−0.0450	0.1653	−0.0509	−0.2113
Tree height (m)	0.0038	−0.2572	0.0897	0.3622	0.2011
DBH (cm)	0.0581	0.6375	−0.0429	−0.4076	0.0823
Trunk circumference (cm)	0.0581	0.6375	−0.0429	−0.4076	0.0823

The results suggest that certain biometric features of trees can serve as robust indicators of their stability. To gain a more precise understanding of tree stability and the associated risks, it is recommended to use instrumental methods in conjunction with visual assessment techniques.

## Discussion

This research involved the examination of 373 of the largest trees located near pathways in urban forests (including 104 hollow-bearing trees and 269 without hollows). Based on the analysis and investigations conducted, it cannot be definitively concluded that the presence of hollows in aged trees significantly contributes to increasing risk within their vicinity. Among all the evaluated indicators for assessing tree stability, only one, the most subjective one reliant on visual assessment, showed an association between the degree of risk and the presence of hollows in trees. Other indicators, grounded in instrumental methods, did not affirm this link.

Risk assessment methods employed by professionals typically have three primary components: likelihood of impact, likelihood of failure, and consequences of failure. Mature and senescent trees in urban sites are subjected to risk minimisation due to the potential repercussions of a tree or part of a tree collapsing, as the size of the tree part expected to fail, and its fall distance are crucial aspects of determining potential damage. Arborists, when asked to rate consequences of failure, tend to select "significant" to "severe" for stems equal to or greater than 29.2 cm in diameter, when the target is a pedestrian, and less stringent 69.2 cm for vehicular targets ^34^. All of the trees surveyed, except one with a stem diameter of 29.98 cm, had stem diameters greater than 29.2 and only 79 out of the 373 surveyed trees had dimensions less than 69.2 cm in diameter. Thus, it can be assumed that these are trees for which the risk class assessed by experts may be inflated due to concerns for pedestrian safety.

In our study, half of the hollow tree group was found in category B (moderate risk), potentially influenced by frequent usage. Trees in class B (moderate) and C (high risk) accounted for 90.3% of the trees in the hollow tree group. The elevated level of risk may be attributed to the physiology of the trees, many of which are in the mature phase, characterised by an increase in trunk thickness, decrease in height growth, and self-cleaning of the crown from shaded branches which can pose a risk to pedestrians. A low proportion of trees in the particularly hazardous (D) group were found- only 2.97% without hollows and 2.88% with hollows. A low proportion of trees in class B may suggest that risk minimisation treatments (e.g., removal of dead branches over a path or crown lowering) are effectively carried out. Our findings indicate a high proportion of hollow trees in classes B and C.

Although mature and senescent trees were examined, which are large in size and thus potentially hazardous, we found 37.5% of hollow trees in risk class B (moderate), and 39.4% in C (high risk class). In the group of trees without hollows, these percentages were 34.5% and 27.1%, respectively. The presence of hollows in aged trees may influence the assignment of a higher risk category during the visual assessment of the tree. The significant correlations between the presence of hollows and the risk class assessment imply that the presence of hollows may indirectly affect the safety classification of a tree, potentially due to uncertainty or other factors that may increase the risk class. This has been observed in studies such as Koeser and Smiley^33^, where parents were 3.4 times more likely to remove a tree given its risk, or participants with children were 1.6 times more likely to select a higher consequence of failure rating than similar participants without children. However, in contrast^50^, found that more frequent visits to the forest resulted in less radical decisions about cutting down old trees.

Our results demonstrate that the Roloff’s scale correlates with the tree risk class. The occurrence of deadwood in tree crowns with impaired vitality is a natural process^51^, but it poses a risk near paths due to the potential of a dead branch falling. Approximately 40% of the trees in both groups were found to be in the R1 class, indicative of good tree vitality, likely due to good site conditions. R2 is a class typical of mature and ageing trees; 35% of trees without a hollow and 31% with a hollow were assigned this class. The lack of significant correlations between the abundance of trees with hollows in each Roloff class (p > 0.5) suggests that the occurrence of hollows in aged trees is not directly related to their vitality as determined by the Roloff scale. Instead, the presence of hollows may be a natural factor related to the tree's age or species. It is likely that tree hollows appear with age, which is not a result of their vitality, but simply a symptom of ageing ^52,53^.

The safety factor results from the SoT and pulling tests did not demonstrate a correlation between the presence of hollow trees and an increase in risk class. The highest proportion of hollow trees (89.42%) was in the low risk group for trunk fracture and uprooting. Interestingly, a slightly higher proportion of trees in this group were found among trees without hollows (94.42%). Similar results were obtained in the study of urban street trees ^13^, where most hollow trees belonged to the low risk class, according to the SoT result. Only a small proportion of trees with hollows, cavities and/or fruiting bodies were classified as hazardous by the SoT method: about 6% at the moderate risk level and 3% at the high risk level. A higher proportion of hollow trees in the high risk trunk fracture category were found: 1.49% from the group of trees without hollows and 3% with hollows. Fewer trees without hollows (3.72%) than with hollows (5.77%) were found in the moderate trunk fracture and uprooting group (category 2). Of the 16 total trees in our study that were classified in group 2 (moderate risk of trunk fracture and uprooting), more of them (62.50%) were trees without hollows, compared to 37.50% of trees with hollows. However, these differences were not statistically significant due to the small sample size.

The majority of the trees with hollows fell into the category of low risk for trunk fracture and uprooting, making up 89% of the hollow trees and 25% of all the trees surveyed. These findings suggest that the presence of hollows in older trees does not directly compromise their stability, as determined by tomograph and load test measurements. In fact, categories associated with moderate to high risk of trunk fracture and uprooting included more trees without hollows than with them.

Contrary to previous studies ^13^, our research revealed a lower percentage (3%, not 9%) of hollow trees in the high risk category. As a result, we propose that decisions regarding tree removal should rely on professional arborist recommendations, bolstered by objective methods such as SoT. Our findings argue against the assumption that cavity presence is a primary indicator of trunk condition.

Our analysis of variance found no significant differences between trees with and without hollows in terms of the safety factor, as determined either by tomography or load test. The absence of differences between trunk mechanical strength and resistance to puncture supports the theory that hollow trees in relatively good physiological condition maintain sufficient mechanical stability through their wood tissue. This aligns with the biomechanical theory of tree function, particularly regarding hollow stems^54–56^ and stays in opposition with common management practices e.g. stating that high risk is connected with stem shell thickness less than 2 in. of sound wood for each 6 inches of stem diameter^57^. This approach seems not consider specifics of mature and especially senescent trees, what can lead to loss of not dangerous, high value trees.

Signs of trunk strengthening are often visible during a visual inspection, and trees can resist decay spread through defence mechanisms^58^. However, we found no evidence of a significantly higher risk level in mature and ancient trees regarding critical wind load failure or SoT trunk investigation.

The results underscore the importance of monitoring the visual barrier zones in hollow trees (especially Wall 4), even in the presence of invasive fungus species infecting the root system. Our analysis showed no significant difference between trees with and without hollows for the SoT safety factor, but a difference was apparent for the load test. Therefore, in addition to assessing vitality, geometry, and mechanical failure reasons, a long-term stability prognosis is crucial.

In general, our findings suggest that the assignment of a higher risk category to hollow trees during visual assessment is likely driven by the diagnostician's responsibility and caution, rather than the actual biomechanical performance of the trees confirming previous founding’s^15,16,31,32,36^. Effective long-term prognosis of tree safety requires a deep understanding of the strategies for reaction-zone creation and the invasiveness of wood decay fungi.

We found differences in Diameter at Breast Height and crown width between trees, but not in tree height, despite the fact that a common response during the mature and ancient phase of trees is top dieback^59^. Notably, trees exhibiting dieback were significantly thicker and had narrower crowns, which, from a biomechanical perspective, improves their stability.

The scale effect, which refers to the maximum dimensions a tree can reach before its stability becomes compromised, was first described in the nineteenth century^60^. This phenomenon arises from the growth optimisation process a tree undergoes, balancing its physiological and mechanical functions with its morphological architecture^61,62^. To maintain mechanical stability, trees can produce modified woody tissue, lower their centre of gravity by increasing the trunk diameter relative to height, or limit crown development. The latter is especially significant as it reduces the transfer of wind loads to the trunk^63–68^.

Statistical relationships were found between the instrumental classification and tree crown width, safety factor and minimum wall of healthy wood, and tree height and DBH. Moreover, hollow trees displayed a slightly different morphological architecture, providing them increased biomechanical stability.

The formation of biomechanical resistance of the stems of hollow trees at a level similar to trees without hollows can be justified, among other things, by adaptive tree growth^27^. According to this theory, during the growth of trees, their growth is optimised in terms of the physiological and mechanical functions performed by the trunk. Metzger^69^ was a forerunner and proponent of the mechanical theory, in which it is assumed that in a growing tree, there is an optimisation of resistance to external forces by maintaining an adequate cross-section of the trunk and height of the tree. During life, the tree optimises its structure to maintain the continuity of the performance of all functions, including mechanical. As a result of a number of external and internal factors, numerous modifications of the tree tissue are produced. They are an essential element of the organism's survival strategy aimed at achieving a compromise between the mechanical properties and hydraulic properties of tree trunks remaining in relation to stress-inducing factors^55,70,71^.

Janzen^56^ emphasises the additional adaptive role of tree hollows as habitats for various organisms, which can enhance soil fertility around the tree base. Therefore, the height of trees, especially those with receding, often hollow, crowns, can be a misleading indicator when categorising trees into high risk classes.

Additionally, hollow trunks are more commonly found in nutrient-poor sites. This suggests that harsh urban habitat conditions may increase the frequency of hollow trunks^72^. If this is not understood, it could lead to the unnecessary elimination of hollow-bearing trees that are valuable to local ecosystems and biodiversity^73–79^.

Predicting the decay is only one of many features and conditions on which failure potential depends, in practice expressed as a risk class. Higher risk of failure in mature trees depends among others: on tree health^29^ presence of decay^30–34^, maintenance history^35–37^, like heavy pruning^37–40^ however, there seems to be a need for special consideration for mature and aged trees. In future risk analyses around trees, it would be helpful to consider the biometric characteristics of trees, especially DBH, tree height, and crown width. Hollow trees were characterised by a more considerable breast height than trees without hollows. This may be due to the fact that they are older, and a natural feature of ageing trees is the presence of hollows.

The coherence of the results of the diagnostic equipment is necessary to provide sufficient information to assess the statics and, ultimately, as our research has shown, the protection of hollow trees.

It can be assumed that aged trees, in order to survive, have optimised the morphological architecture and structure of the woody tissue, as a result of which their biomechanical stability is not significantly lower than trees with trunks without cavities. Of course, this thesis cannot be applied to all trees with cavities (hollows) and consider them safe in advance. However, the authors wish to emphasise that hollow trees should not be considered particularly dangerous and designated for removal without a detailed analysis, based on visual assessment alone.

It should be noted that instrumental methods, which verify the often critical subjective visual assessment, are of limited use in forest areas due to the practical aspect (large population of trees). For this reason, our research is exploratory and for forest land managers to indicate this aspect of the protection of hollow trees.

Consideration should also be given to revising methods of visual assessment of trees so that in the future, subjectivity related to the insufficient experience of the assessor or due to fear of liability and potential legal consequences in the event of a tree fall can be eliminated as much as possible.

### Supplementary Information

Supplementary Information.

## Data Availability

The datasets generated during and/or analysed during the current study are available from the corresponding author upon reasonable request.
