# Supplementary figures and images for: Risk assessment of hollow-bearing trees in urban forests
Source: Sci Rep. 2023 Dec 14;13:22214. doi: 10.1038/s41598-023-49419-0 (PMC10721860; doi:10.1038/s41598-023-49419-0)

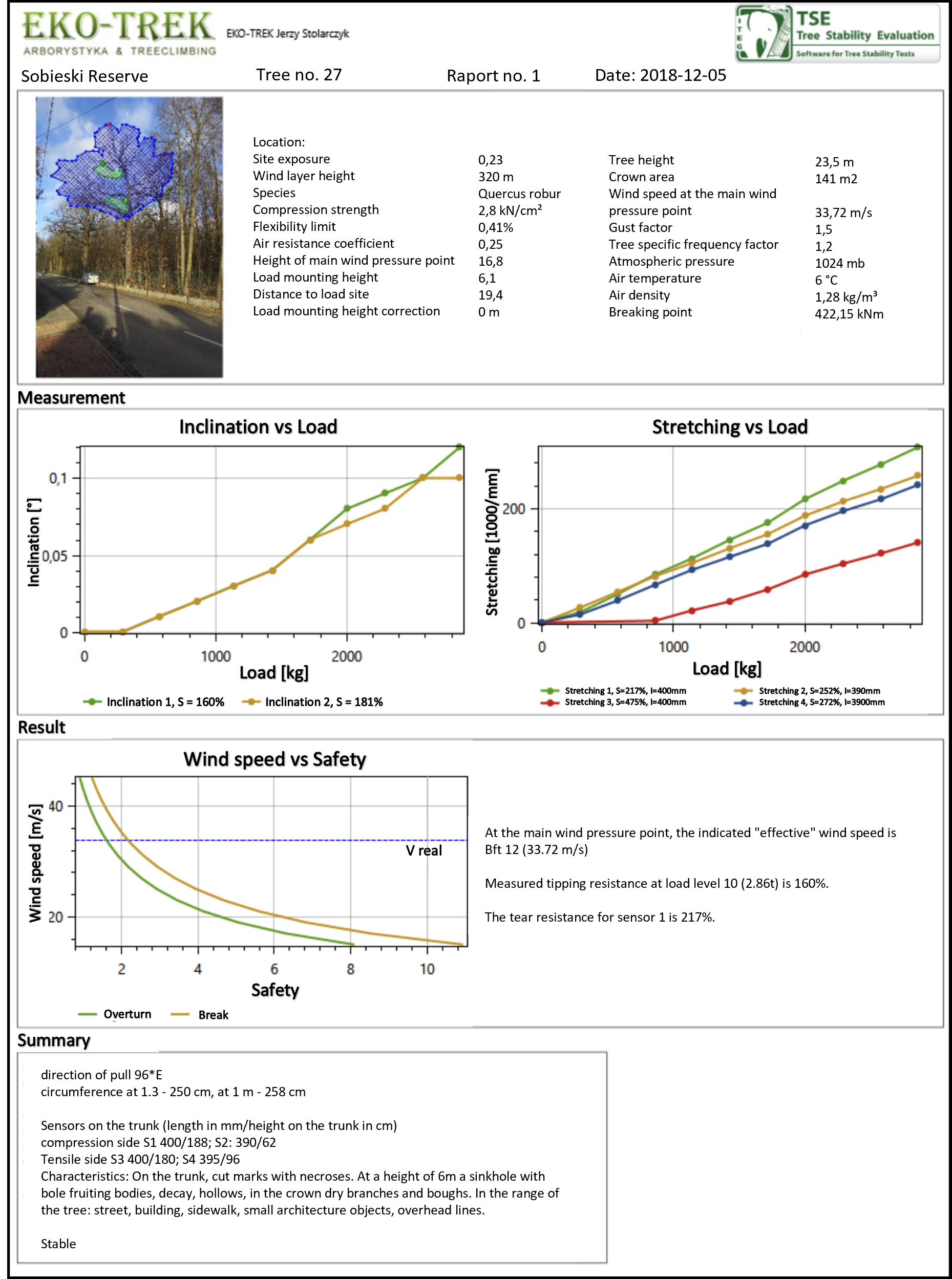

Supplement: Supplementary file 1 — Supplementary Information. [file 41598_2023_49419_MOESM1_ESM.png]
